# Dietary changes as a risk factor and remedy for meditation-related challenges

**DOI:** 10.3389/fnut.2025.1651167

**Published:** 2025-10-23

**Authors:** Josie R. Lee, Nicholas K. Canby, David J. Cooper, Jared R. Lindahl, Willoughby B. Britton

**Affiliations:** ^1^Department of Psychiatry and Human Behavior, Warren Alpert Medical School, Brown University, Providence, RI, United States; ^2^Department of Religious Studies, Brown University, Providence, RI, United States

**Keywords:** diet, meditation, meditation-related challenges, fasting, grounding, nutrition

## Abstract

**Objectives:**

Recent research has documented a range of challenging, distressing, or impairing experiences that can result from Buddhist meditation practices (Lindahl et al.). The present study investigates the impact of dietary changes on the trajectories of Western Buddhist meditators who reported meditation-related challenges.

**Methods:**

Interviews were conducted with 68 Western Buddhist meditators and 33 meditation experts (teachers and clinicians).

**Results:**

Thematic analysis resulted in the following observations: (1) dietary restrictions could be a risk factor for the development of meditation-related challenges; (2) a loss of appetite or lack of eating was often an exacerbating factor and diagnostic indicator of more severe distress when meditation-related challenges were already occurring; and (3) diet-related remedies, such as eating “heavy” foods and meat, were often described as helpful and associated with “grounding” effects for meditators-in-distress.

**Conclusion:**

This study highlights the importance of considering diet-related factors as both risk factors and remedies for meditation-related challenges and suggests possible implications for research and practice.

## 1 Introduction

Over the past several decades, Buddhist meditation and Buddhist-derived meditation practices such as mindfulness have become popular in the United States and other Western countries, where they have been increasingly secularized and integrated with Western psychology (1, 77). Although a wide range of mental and physical health benefits of meditation practices have been documented (2, 3), unusual, challenging, distressing, and even impairing experiences have also been reported (4–8). Challenging meditation-related experiences are described in texts across several Buddhist traditions and have been found to occur in both clinical and religious settings in contemporary Western cultural contexts (5, 9, 10).

Recent research from the Var*ieties of Contemplative Experience* project has focused on documenting and describing the range of meditation-related challenges, their associated causes and influencing factors, how they are interpreted, and the strategies used to mitigate and manage them. Based upon phenomenological interviews with meditation practitioners and meditation experts (teachers and clinicians), Lindahl et al. (8) documented 59 distinct types of meditation-related challenges spanning seven domains (cognitive, perceptual, affective, somatic, conative, sense of self, and social) and 26 influencing factors spanning four domains (practitioner-related, practice-related, relationships, and health behaviors). As part of the health behavior domain, dietary changes were identified by both meditation practitioners and meditation experts as an influencing factor for the trajectory and/or severity of meditation-related challenges. The present study seeks to unpack the relationship between dietary changes and meditation-related challenges in Western Buddhist meditators in greater depth.

Dietary restrictions involving transient or irregular acts of deprivation in the kind or amount of food intake are an extremely common element of religious traditions and are often associated with religious rituals (11, 12). Religious adherents engage with them for numerous reasons, such as maintaining ethical conduct, preserving bodily and spiritual purity, or performing religious identity (13, 14). Monastics, contemplatives, mystics and other dedicated practitioners of asceticism engage in ongoing or long-term dietary restrictions, including fasting, which has both positive and challenging physiological and psychological impacts (15). In fact, both fasting and involuntary appetitive changes are associated with spiritual development, spiritual attainments, and saintliness (11, 16) as well as concerning psychological distress including first-episode psychosis and mania (17, 18).

Food-related practices and dietary restrictions vary considerably by Buddhist lineage, geographical region, and historical era (19, 20). It is beyond the scope of this paper to offer a comprehensive discussion of food-related practices even among contemporary Buddhist traditions in the West (21, 22). However, it is worth noting that for the Western Buddhist meditators who are the focus of this analysis, changes to their dietary practices often occurred either in the context of intensive meditation retreats, where dietary restrictions such as vegetarianism and/or reductions in food intake are common, or as a result of navigating other meditation-related challenges that arose during retreats or through daily practice.

Diet has a well-established relationship with mental health (17, 23–25) From a scientific perspective, the nervous system relies on nutrients supplied by food such that the timing, quantity, and composition of dietary intake can impact short-term mood, cognition, and emotion regulation (26, 27) and have long-term effects on mental health (25). This relationship is bidirectional, as mood can also impact appetite and dietary intake (28–31). Furthermore, dietary changes can be used to treat or prevent mental health disorders. Whole dietary patterns (e.g., the Mediterranean-Type) and supplements (e.g., omega-3 fatty acids, vitamins, amino acids) have been used to effectively treat and prevent relapses of generalized anxiety disorder, major depressive disorder, bipolar disorder and schizophrenia [for reviews, see (17, 32–35)].

In some East Asian medical systems, such as Traditional Chinese and Tibetan Medicine, food is considered to influence the flow of “energy” (e.g., Chn. *qi,* Tib. *rlung*), which affects different functions of the mind and body (24, 36, 37). Though explanatory frameworks for health and illness differ across traditions, poor diet is considered a contributing factor to a range of mental health challenges (24). Importantly, mental stress (e.g., tension, worry or low mood) can similarly disturb the flow of “energy” and cause a variety of symptoms including poor digestion and/or a loss of appetite (24, 38). To restore the balance and alleviate mental and physical stress, traditional Tibetan and Chinese medical practitioners prescribe foods that are characterized by various qualities including taste, temperature, density, and color (36, 39).

Less is known about the relationship between contemplative or religious practices, dietary intake, and subsequent mental health. In addition to their concomitant ethical and cultural systems, it is possible that certain contemplative techniques alone can precipitate changes in eating behavior. For example, Buddhist-derived mindfulness meditation training has been associated with reduced food cravings and emotional-eating among individuals with a tendency to stress eat (40). Additionally, dietary restrictions in religious contexts have been associated with altered and/or unusual states of mind (41). For example, Baha’i fasting has been associated with increases in mystical experience and mindfulness (42) and fasting during Ramadan has been linked to a higher rate of relapse in bipolar disorder (43, 44). To our knowledge, there is a lack of interdisciplinary research on the interaction between dietary changes and Buddhist meditation practice in contemporary Western contexts.

The present study aims to examine the influence of dietary changes on trajectories of meditation-related challenges among contemporary Western Buddhist meditators. Using a secondary analysis of the diet-related changes reported by meditators and meditation experts in the Var*ieties of Contemplative Experience* project, we investigated the role of dietary changes as a risk factor, remedy, or co-occurring symptom before, during, and after meditation-related challenges. We also examined expert perspectives on monitoring and altering students’ diets in response to unusual or challenging experiences.

## 2 Methods

### 2.1 Study sample

The present study includes data collected for the *Varieties of Contemplative Experience* (VCE) research project, which is a mixed-methods study on meditation-related challenges reported by Buddhist practitioners in the West (8). The VCE study team interviewed 68 Western Buddhist meditation practitioners who could describe challenging, difficult, distressing, or functionally impairing experiences that they associated with meditation practice, and 33 meditation experts (teachers and clinicians) who had worked with practitioners experiencing these challenges. Following interviews, practitioners were asked to complete a quantitative online survey that assessed causality and further queried demographics and influencing factors. Of the 68 practitioners interviewed, 39 spontaneously mentioned dietary or appetitive changes in their interviews, and 37 indicated in the follow-up online survey that they had tried dietary changes as a remedy for their meditation-related challenges. Eleven participants mentioned dietary or appetitive changes *only* during the interview, 11 mentioned dietary remedies *only* in the survey, and 28 participants mentioned dietary changes in both data sources. In total, 50 of the 68 practitioners (74%) referenced dietary changes in at least one data source. See Table 1. We take these practitioners as the study sample for the present paper.

**Table 1:** VCE study participant references to diet by data source.

	*N* = 68	*%*
Mentioned dietary or appetitive changes in interviews	39	57
Reported dietary changes in follow-up survey	37	54
Reported dietary changes in either data source	50	74
Reported in interviews only	11	16
Reported in survey only	12	18
Reported in both data sources	28	41

### 2.2 Participant demographics

The demographics of the practitioners in this sample and in the larger VCE project are detailed in Table 2. The demographic distributions were comparable between the full sample and the subsample.

**Table 2:** Practitioner demographics and characteristics: full sample and subsample.

	All VCE practitioners		VCE practitioners who reported dietary changes	
	*N* = 68	*%*	*N* = 50	*%*
Gender				
Female	31	46	24	48
Male	37	54	26	52
Nationality				
American	56	82	41	82
Canadian	3	4	2	4
Middle Eastern	1	1	1	2
British	3	4	3	6
Other European	4	6	3	6
Mexican	1	1		
Sexual Orientation				
Heterosexual	54	79	41	82
Gay/Lesbian	6	9	3	6
Bisexual	3	4	3	6
Other	5	7	3	6
Race				
White	60	88	45	90
Native American or Alaska Native	1	1	1	2
Black	0	0	0	0
Asian	0	0	0	0
More than one race	3	4	2	4
Other Race	4	6	2	4
Ethnicity				
Hispanic	3	4	2	4
Non-Hispanic	61	90	46	92
Prefer not to answer	4	6	2	4
Practice tradition				
Goenka	8	12	10	20
Theravāda	20	29	13	26
Tibetan	20	29	13	26
Zen	20	29	14	28
Lifetime practice hours				
0–100	3	4	2	4
100–500	2	3	1	2
500–1,000	3	4	3	6
1,000–5,000	16	24	14	28
5,000–10,000	16	24	10	20
10,000	28	41	20	40
Symptom duration				
Less than 1 week	4	6	1	2
1 week to 1 month	8	12	5	10
1 to 6 months	18	26	11	22
6 to 12 months	5	7	5	10
1 to 3 years	13	19	9	18
3 to 6 years	7	10	7	14
6 to 10 years	2	3	2	4
More than 10 years	11	16	10	20

### 2.3 Procedure

In semi-structured VCE interviews, practitioners were asked to describe their meditation-related challenges, how these experiences were interpreted by themselves and others, and what responses and remedies were helpful or unhelpful for navigating their challenges. As mentioned above, an online follow-up survey was used to collect additional information. This survey explicitly asked whether participants tried changing their diet as a remedy. It did not explicitly ask about dietary changes as a risk factor. Meditation experts were also interviewed on the types of challenges that they had seen in their students and/or clients, as well as how they interpreted and managed such challenges. A team of researchers transcribed interviews and used open coding techniques described by Corbin and Strauss (45) to code phenomenology, risk factors and remedies, and determining need for intervention. To ensure interrater reliability, a team of three coders re-coded 15 % of practitioner and expert interviews and reached agreement that ranged from 93 to 100% (weighted kappa = 0.70 and 0.56 respectively).

### 2.4 Analysis

For the present qualitative analysis, all VCE interviews in which appetitive or dietary changes were coded were identified. Then, using the thematic analysis method outlined in Braun and Clarke (46), the primary author read through all identified interviews and generated 20 codes. All authors met to discuss the codes and create themes. Once themes were established, the first author recoded the data to evaluate the new theme structure. Multiple authors (JL, NC, DC, and JL) continued to meet to iteratively revise the themes. The final structure of themes was established and defined once all authors reached agreement. Lastly, representative quotes were selected to illustrate the themes in the data.

Quantitative information on demographics and participant ratings of dietary changes as a remedy for meditation-related challenges were included in the present analysis from the follow-up survey. Data that was missing from the follow-up survey was imputed by the research team when the information was available in the qualitative interviews.

## 3 Results

### 3.1 Primary themes

#### 3.1.1 Restricted diet prior to meditation-related challenges

Many practitioners in the Var*ieties of Contemplative Experience* study reported that they reduced their food intake and/or stopped eating animal meat as a part of their contemplative practice prior to the development of their meditation-related challenges. Practitioners who were living in a retreat center, remote location, or monastery often described eating “slightly less” than they normally would. One meditator described this as part of a “normal monastic diet,” in which one is instructed to “eat and drink moderately.” However, a subset of meditators reported a significant reduction in food intake during their time at a retreat center or monastery. Some such meditators were not served dinner or were served food only in the morning (two meals before noon).

Meditators described mixed reactions to these reductions in food intake. Though eating slightly less was generally not viewed by practitioners as concerning or problematic, some practitioners described experiencing significant challenges when stricter dietary constraints were imposed by their practice context. One described feeling “pretty peaceful” at the time, while another remembered “intense hunger pains.” A few meditators who were only served food in the morning reported losing a considerable amount of weight. One meditator, who said he looked “like death” after a five-month retreat in Burma, recounted: “I remember talking to one of the monks and I was like, ‘I’m leaving, this sucks. I’m done.’ I had lost so much weight […]; I weighed […] 120 pounds.” Around this time, he described not being able to “be aware of a concept” and feeling physically weak. Although he ended up deciding to stay, finding that his mind became “more steady” over time, eventually he became very sick and returned to the United States out of concern for his health.

Many meditators also shared that they were vegetarian around the time of their challenges or that they had stopped eating animal meat as a part of their practice. Some described a link between a light vegetarian diet and meditation-related experiences that involved feelings of positive affect, weightlessness, and emotional lability. One practitioner at a Tibetan Buddhist nunnery recalled that:

I also was a vegetarian back then and I was […] basically subsisting on rice and weird cooked vegetables. So I think I was probably super protein-deficient. […] But I did lose a ton of weight, and I just remember it was kind of blissful at times, because if you do that many prostrations and you get into the rhythm, and you know you are doing the mantra and the prostrations right? And I can just remember feeling like I was almost like flying, which is kind of nice. […] But that’s the time I remember having really intense [feelings …], and I would just bawl for like four hours.

Other practitioners drew a connection between vegetarian diets and more distressing experiences. For example, one man who had been practicing various Tibetan forms of meditation for several years developed “what Tibetans call a *rlung* disorder,” which he described as feeling “wired,” like his chest was being squeezed “between two metal plates” and like he had drunk “50 cups of coffee.” He recalled, “I think one of the first times that [the *rlung* disorder] got really bad was actually when I was almost being a vegetarian—like not much meat.”

Several practitioners and experts described their views on how vegetarianism can directly influence meditation-related experiences. One Tibetan Buddhist teacher shared his observation that energy-related challenges seemed to be more common among vegetarian students who were not “well-nourished”:

You know, the sort of classic grounded energy is somewhere in the midsection. You’re grounded to the Earth, you are grounded with others. You’re embodied. You know what is going on in any given moment. And your energy can begin to rise. And this happens particularly when people aren’t well-nourished, if they are vegetarians—bad vegetarians, not all vegetarians. The energy begins to rise and so it rises up above that. You lose your connection with the Earth.

#### 3.1.2 Appetitive changes and co-occurring phenomenology

Not all diet-related changes reported in the Var*ieties of Contemplative Experience* study were imposed by a practitioner’s context. In fact, many practitioners had access to a balanced diet but experienced a significant loss of appetite. Oftentimes appetitive changes co-occurred with other unusual or challenging meditation-related experiences, though it was not always clear if and how these co-occurring phenomena were related. Several meditators and teachers suggested that a loss of appetite and reduction in food intake could exacerbate other challenging or unusual meditation-related experiences.

Many meditators reported a reduction in appetite alongside changes in perception or sense of self —especially perceptual alterations in time or space. For example, one practitioner remembered losing her appetite during her first silent *vipassanā* retreat as she entered into a “flow” state in which she was no longer aware of “linear concepts like time.” This practitioner began to eat very little and “would just sit up all night meditating outside and then start to go to bed when the sun would start to come up.” Eventually she was hospitalized by the retreat staff after experiencing extreme fear followed by a sense of being “one with everything,” a loss of identity, delusional beliefs, and changes in visual perception, which collectively disrupted her ability to follow retreat protocols or communicate normally. Another meditator who had a daily Zen practice also described losing her appetite following a moment during meditation in which “everything fell away.” This practitioner recalled a sense that time had stopped and there was no “sense of me,” while “everything [was] just floating in this most gloriously beautiful way.” Given that she began “to not eat or sleep very much,” her Zen teacher decided that she should move in to the meditation center and be monitored closely. Despite this support, her perceptual and appetitive changes persisted, and she went on to lose a significant amount of weight. For approximately a year and a half these symptoms impaired her ability to care for her children and to work.

A handful of practitioners described developing gastrointestinal distress because of the emotional or mental stress they experienced through meditation. The digestive challenges that meditators reported varied, but in all cases, they affected the meditators’ ability or desire to eat. For example, one practitioner developed a host of somatic challenges, including gastrointestinal distress, while engaging with tantric practices that involved “using visualizations of channels and energies and moving energy through [her] imagination [and] body.” She shared that at the time she “did not want to eat at all because [she] was on the toilet all the time.” Similarly, another practitioner shared how he became unable to digest food while practicing at a series of *vipassanā* retreats in Burma:

I began losing weight—my ability to digest food also stopped. So even if I ate just a little bit of food it felt like it would sit in my stomach for hours and then I would not be hungry for the next meal. And I lost a lot of weight. So maybe the next three months was a time where this sense of exhaustion got stronger and stronger and more persistent. And my ability to bioregulate began to fall apart in that I was exhausted through the day, I was restless at night, I could not digest my food, and my mind was restless and my body was tired, and I was losing weight and feeling weak.

This practitioner returned to the United States out of concern for his health and continued to experience extreme fatigue for 4–6 years. In his recovery he interpreted his symptoms as “physical manifestations” of “emotional and mental patterns” that he “broke apart” while working with a teacher who had a particularly “aggressive” approach. His mental and physical health improved “slowly” as he learned “different strategies for how to be in the world and deal with stressful situations.”

Several meditators also reported a loss of appetite in relation to energy-like somatic experiences [ELSEs; see (47) for an in-depth description of this topic]. One practitioner on a *vipassanā* retreat began to experience a continuous sense of “energy running,” including “the sensation of tons of energy […] shooting out through the palms of [her] hands.” When teachers became concerned about her, she revealed that she had not “been eating and sleeping that much because [she] just did not feel like it—[she] just had all this energy going.” Her teachers responded, “Yeah, that will really aggravate this.” Similarly, a Tibetan Buddhist practitioner on a six-month solo retreat was engaging in an inner-heat practice (Tib. *gtum mo*) that intentionally worked with somatic energy in the body when she began to experience the energy “building, building, building.” As the energy “amped up,” she had a wide range of unusual experiences, including a sense of being directed and “inhabited” by a disembodied teacher who gave her often-strange instructions, including about what to eat. Amidst this, she “wasn’t eating very much” and was “losing lots of weight.” When she finally saw her human teacher three months in, he immediately told her to let go of the idea of the disembodied “guru,” gave her some energy balancing practices, and “got [her] eating” and sleeping regularly. With this perspective, she later reassessed some of the things she had been doing as “crazy.”

Finally, many practitioners who reported appetitive or weight changes also reported co-occurring sleeplessness. Participants often mentioned these phenomena in the same breath, suggesting that they perceived a lack of eating and sleeping to be connected or affected by the same underlying mechanism. A few participants mentioned these phenomena in reference to a self-described “manic” state. For example, one Theravāda Buddhist practitioner described “getting into a bit of manic-ness” in reference to “periods of immense clarity and freedom” in which she was “barely” eating or sleeping. Despite the reference to mania, this practitioner interpreted her experiences as a stage of insight and did not seek medical intervention. Another practitioner described not eating or sleeping “for long periods of time” while experiencing a growing sense of mania that came on following a *zazen* self-retreat in Japan. This practitioner shared that he began experiencing racing thoughts during his meditation practice that he perceived to be highly creative, coupled with a sense of specialness, pride, and a desire to write for long periods of time. Although he described this state as having “seductive” qualities, this practitioner became increasingly destabilized until eventually his creative thoughts and inflated mood abruptly ended and were replaced by a sense of unbearable “existential dread.” Instead of seeking professional support, this practitioner lived with a friend for two weeks to “ride out the storm.”

#### 3.1.3 Dietary changes as a factor in differential diagnosis

Practitioners’ dietary changes were also discussed by meditation teachers when differentiating between mental health crises that required intervention and expected stages of practice. Some teachers described monitoring students’ food intake when determining the potential causes and severity of their students’ challenges and when determining what form of intervention they deemed necessary. An overwhelming consensus from meditation teachers across traditions was that not eating enough (as understood within the practice context) was a “real red flag” or a “recipe for disaster.” One Theravāda Buddhist teacher described how students who are not eating or sleeping can get “wound up in an ecstatic way.” As a result, this teacher reported that whenever a student comes to him in “a bit of a manic way,” he explicitly asks the student how much, and what, they are eating. In cases in which a student is eating “almost nothing,” he recommends that the student focus less on their meditation practice and take time to eat, sleep, and walk.

In some cases, meditation teachers implied that a loss of appetite was a byproduct of the student’s challenge. A handful of meditation teachers reported instances in which appetitive changes were symptomatic of a more serious mental health challenge, such as psychosis. One Theravāda Buddhist teacher recounted the trajectory of a student who came on retreat very eager to progress in her meditation practice but then began experiencing auditory hallucinations, behaved disruptively, and stopped eating. Despite the teacher’s efforts to feed the student, the student did not improve, which led the retreat staff to seek psychiatric support.

#### 3.1.4 Diet-related remedies to meditation-related challenges

A variety of diet-related remedies were discussed, attempted, and recommended by both meditation practitioners and experts in response to meditation-related challenges. The quantitative survey revealed that 37 practitioners interviewed for the Var*ieties of Contemplative Experience* study (*N* = 68) tried changing their diet in response to meditation-related challenges. These practitioners reported mixed effects: 46% of them experienced no impact and 54% of them found dietary changes to be helpful. No practitioners reported dietary changes to be harmful. Among the 20 participants who found dietary changes to be helpful, ratings ranged from somewhat helpful (65%), to moderately helpful (15%), to very helpful (20%). See Table 3. The diet-related remedies that the sample described drew from several medical traditions (Chinese, Tibetan, and allopathic medicine), and included remedies such as consuming more food or alcohol; eating a “rainbow” diet; eating eggs, sugar, or “heavy” foods; or eating animal meat.

**Table 3:** Participant survey-reported ratings of diet change as a remedy for meditation-related challenges.

	*N* = 37	%
No impact	17	46
Harmful	0	0
Helpful	20	54
Somewhat helpful	13	35
Moderately helpful	3	8
Very helpful	4	11

Overall, we found many participants used the term “grounding” to describe their intention and/or phenomenological experience of diet-related remedies. Meditation experts from different Buddhist traditions described eating as a “grounding” intervention for students presenting with challenges. Some experts believed that their students’ challenges were related to a reduction of food intake and found that a “low-threshold” remedy for these students was to give them a meal. A handful of meditators described changing their diet as a response to feeling “too high,” as if they had lost touch with the ground. Experts and meditators reported that root vegetables, small quantities of beer or wine, and meat were helpful in such circumstances because they bring energy “down.” For example, one *vipassanā* meditator reporting energy-like somatic experiences (ELSEs) and prolonged perceptual hypersensitivity described receiving helpful advice from a Tibetan nun to “drink alcohol, especially beer—it’s going to ground you. Eat lots of meat.” Participants reported mixed perspectives on sugar, however. One Zen practitioner who reported an unusual energetic experience during a *sesshin* said that her teacher suggested that she eat a “hamburger or […] a couple of candy bars,” because they were considered “spiritual depressants.” This meditator took her teacher’s advice, ate a couple of candy bars, and was able to continue with the *sesshin*. In contrast, another meditator reported avoiding sugar, as well as caffeine, because he found them to “exacerbate” painful somatic energy.

Several meditators, especially those who dealt with energy-like somatic experiences (ELSEs) or *rlung* disorders (a diagnosis in Tibetan medicine that is related to an imbalance of vital energy), found re-incorporating animal meat—chicken, fish and, in particular, red meat—to be grounding. In fact, a handful of practitioners who had been vegetarian for several years described craving meat during the most challenging points of their experiences. One meditator who reported feeling “wired,” “dizzy” and unable to concentrate during his daily practice recounted amazement after taking a Tibetan doctor’s advice to eat meat:

Basically then I realized how much I was craving meat. And also, literally the moment I was eating meat—like after 10 to 20 s—I actually did feel a lot better. Like, the wired-ness stopped almost completely. […].

Other practitioners reported that eating meat had a slower remedial effect or that eating meat was one component of a combined “therapy structure” that included psychotropic medications or other remedies. One meditator, who felt like her mind was “shattered” after a six-week *vipassanā* retreat, was told by her teacher that: “You’re going to need to eat meat. And I want you to garden. And I want you to do exercise every day and take sleeping pills and sleep. And take your antipsychotic medication.” This practitioner followed her teacher’s regimen for a year and her symptoms improved over time.

Some experts recommended that their students eat, not necessarily because they were not eating enough, but because they considered the *act* of eating to have grounding effects. Eating, exercising, gardening, and sex were often described as grounding by both experts and practitioners due to their connection to physical, sensory, and embodied aspects of experience. One meditator described how eating “heavier foods” helped him to remediate “a sense of disembodiment or disconnectedness” that he started to experience while on a three-year Tibetan Buddhist retreat. Similarly, another meditator shared that if she ever started to feel “light and fluffy,” she would ground by eating “hot heavy food.” Furthermore, one Zen practitioner and teacher described eating, along with exercise, to be grounding and helpful in alleviating his symptoms because it focused on the “gross body” rather than rather than on the energies of the subtle body, which had been the focus of his *prāṇāyāma* practice. Eating was also described by some participants as leading to emotionally grounding effects. One woman who described not “feeling grounded and safe” due to distressing changes to her sense of self, reported that eating certain foods that reminded her of positive childhood memories could be “grounding” and make her “feel good.”

## 4 Discussion

This paper outlines various ways in which contemporary Western Buddhist meditators described dietary changes playing a role in the incidence, symptomology, and recovery from meditation-related challenges. The analysis yielded the following themes: (1) a reduction in food intake and meat consumption was perceived to increase the likelihood of meditation-related challenges; (2) a loss of appetite was reported to be a symptom of meditation-related challenges that could exacerbate such challenges when accompanied by not eating enough; (3) not eating enough was considered a key factor in differentiating normal or normative meditation-related challenges from those requiring support or intervention; and (4) dietary changes were often considered to be a helpful remedy for meditation-related challenges, particularly those described as “grounding.” Given that there is little prior research on the role of dietary changes in contemplative practice trajectories or meditation-related challenges more specifically, these findings introduce a number of novel hypotheses for future investigation.

### 4.1 Dietary restrictions as a risk factor

Participants in the present study characterized dietary restrictions, such as a significant reduction in food intake or meat consumption, as a potential risk factor for the development of meditation-related challenges or exacerbating factor when challenges were already present. While the current study cannot infer causality, our study generated hypotheses regarding three potential pathways through which dietary restrictions could affect meditation-related challenges: (1) a link between reduced food consumption and altered or “ungrounded” states of consciousness (including mania and psychosis); (2) a link between reduced food consumption and acute changes in cognitive and emotional functioning; and (3) micronutrient deficiencies especially related to a lack of meat consumption.

First, fasting, in and out of religious contexts, has been associated with a variety of altered and challenging states of consciousness, ranging from euphoria, religious experience, and “self-transcendence” (41, 48–50) to first-episode psychosis and mania (17, 18). Some participants in the present study who reported eating significantly less than their normal diet experienced positive and pleasant phenomenological shifts while others reported more concerning, and potentially dangerous, meditation-related challenges. The evidence that some of these states may be perceived as pleasant or desirable while others may be destabilizing or “ungrounding” is consistent with a perspective in which processes that are adaptive in moderation become maladaptive when taken to an extreme (51).

Another way in which restrictive dieting may be related to meditation-related challenges is due to its association with low glucose levels and short-term negative emotionality and cognitive deficits. Short term fasting has been found to be associated with decreased subjective mood, increased irritability, and reduced performance on cognitive and attentional tasks (52–54), which may be explained by low glucose levels in the brain. Low glucose levels are associated with reduced self-control, cognitive performance, and emotion regulation as well as increased aggression and impulsivity (55–57). Furthermore, given that eating can function as a form of emotion regulation (30), restrictive dieting may inhibit access to emotion regulation strategies. Therefore, we hypothesize that these factors could contribute to the occurrence of meditation-related challenges by inhibiting cognitive and emotional self-regulation.

Finally, participants’ meditation-related challenges could be related to micronutrient deficiencies resulting from restrictive dieting. Many meditation practitioners in the present study reported that they were not consuming animal meat at the time of their unusual or challenging experiences and some practitioners drew a connection between their vegetarianism and changes to their affect, mood, and somatic experience. Vegetarian and vegan diets, if not properly balanced, can increase the risk of deficiencies in nutrients such as protein, omega-3 fatty acids and vitamin B12, which can result in mental health-related symptoms (32, 58–61). Some of their reports of significant reduction in food intake, cognitive challenges, fatigue, and changes in sense of embodiment suggest future work exploring this nutrient-deficiency hypothesis is warranted (60, 62).

### 4.2 Loss of appetite as co-occurring symptom and diagnostic factor

Practitioners and experts described a loss of appetite and an ensuing lack of dietary intake as an exacerbating factor when individuals were already experiencing meditation-related challenges, as well as a diagnostic indicator of a greater degree of destabilization. At times, practitioners described losing interest in their bodily needs (such as eating and sleeping) during experiences in which they lost track of space, time, their sense of self, and/or their embodied existence in physical reality. At other times, practitioners experienced reductions in their appetite or eating as a result of their bodily experience, such as a felt sense of gastrointestinal distress or energy-like somatic symptoms. In all of these cases, a lack of eating, at times in combination with a lack of sleeping, was described as exacerbating preexisting symptoms and leading to greater destabilization. This is in alignment with research that indicates that highly restrictive eating and weight loss can lead to severe forms of psychological distress (63).

Experts similarly agreed that a lack of eating was indicative of more serious forms of destabilization and, as a result, was a primary target for assessment and early intervention. Poor appetite and/or overeating are criteria listed for several disorders in the Diagnostic and Statistical Manual of Mental Disorders (DSM), thus mental health providers are advised to monitor significant appetitive or dietary changes when diagnosing eating, anxiety, depressive, bipolar, and substance-use-related disorders (64). Loss of appetite has been found to be associated with heightened stress and stimulant-induced concentration (28, 29), a lack of eating and sleeping may especially be indicative of states of nervous system hyperarousal, which can be induced by meditation and are theorized to play a role in many meditation-related challenges (65). Historical and modern case studies further suggest that dietary patterns are important to consider because religious fasting practices can develop into eating disorders such as orthorexia nervosa and anorexia nervosa (66, 67), the latter of which can be fatal. Finally, a decreased need for sleep, an elevated or expansive mood, and feelings of energy in the body are symptoms characteristic of mania (64), which can be triggered by fasting (43, 44). In sum, a loss of appetite and lack of eating or sleeping are likely to be important indicators of meditation-related dysregulation and risk that meditation teachers should consider monitoring closely.

### 4.3 Dietary remedies for meditation-related challenges

Practitioners and experts described various dietary changes as helpful in remedying their meditation-related challenges. Importantly, over half of practitioners who tried changing their diet in response to meditation-related challenges reported it to be helpful while none found it to be harmful. This indicates that particular dietary changes may be a somewhat effective early intervention for meditation-related challenges with few associated downsides.

Meat, heavy foods (e.g., high fat, high protein), root vegetables, and alcohol were particularly described as being helpful. Many practitioners sought the help of traditional medicine practitioners; thus, it is unsurprising that the foods that participants described align with recommended foods for treating a *rlung* disturbance (24). In Tibetan medicine, *rlung* disturbances are treated with herbal medicine, physical therapy, and a diet that is “high in protein” and comprised of “nutritious” foods, such as “oil, butter, mutton with vegetables, soups, porridge, hot milk and a little alcohol” (39). While these diet-related remedies have little scientific precedent to date, they may be related to some of the pathways that we hypothesized to be involved in diet-related risk factors. For example, it is possible that dietary changes (1) reverse the psychological effects of fasting by increased food consumption and (2) reverse the physical and psychological effects of micronutrient depletion through micronutrient repletion. In addition, we hypothesize that dietary-remedies reported by participants may have been helpful as result of (3) the biobehavioral effects of calorically-dense comfort food and (4) the psychological and emotional effects of comfort eating.

Micronutrient repletion refers to the reversal of micronutrient deficiencies through whole foods or supplements (33, 68). Both omega-3 polyunsaturated fatty acids (PUFASs) and vitamin B supplements can be effective in repleting micronutrient deficiencies and treating a range of related psychiatric symptoms, including anxiety, cognitive disturbances, and psychosis (33, 69, 70). Considering that animal meat contains several essential micronutrients such as protein, B-vitamins, iron, and omega-3 fatty acids (68), we hypothesize that some participants’ recoveries were in part the result of micronutrient repletion. Importantly, there is evidence to suggest that micronutrient supplementation is more effective when done as an early intervention (33) and when addressing a very poor diet (71).

Animal meat, heavy foods, and alcohol might have also been helpful for participants due to their short-term effects on stress reactivity and arousal. Studies have found that rats and/or humans fed “comfort food,” defined as foods high in fat and sugar, release fewer stress hormones and exhibit fewer emotional and behavioral responses when exposed to stressful or emotionally intense environments (30, 72–74). Experimental studies suggest, however, that such comfort foods have only short-term effects in reducing negative affect and dampening the stress response (75). Considering that meditation-related adverse effects have been conceptualized as relating to increased and/or dysregulated arousal of the nervous system (5, 65), it is possible that consuming food and beverages that dampen the physiological stress response could provide participants with short-term comfort or relief.

Finally, participants often described helpful diet-related remedies using the term “grounding.” This term was used to describe a range of phenomenological experiences—from remediating a sense of “disembodiment” by helping participants focus their attention on their physical body, to soothing energy-like somatic symptoms, to eliciting positive memories. The term “grounding” points to an existential fact of human existence: that being alive means depending on the ground beneath us. This fact, some argue, is a fundamental prerequisite for ontological security (76). Thus, if participants associated dietary changes with a return to a familiar, dependable, and secure physical, affective, and/or cognitive state, it may be reasonable for them to describe that diet as “grounding.”

### 4.4 Implications

Our findings highlight the importance of considering dietary practices when engaging in or researching contemplative practices, as well as seeking to prevent or respond to meditation-related challenges or other altered-state experiences. In particular, practitioners may benefit from information regarding the risks of retreats that involve significant dietary restrictions, while teachers may benefit from routinely asking their students about dietary and sleep patterns so that potential challenges can be prevented or addressed early on. Furthermore, practitioners already experiencing challenges may benefit from exploring diet-related remedies while researchers should continue to investigate these remedies as potentially low-cost approaches to treating meditation-related challenges. Retreat centers may benefit from offering a wider range of nutritional choices, including animal-based protein and other grounding foods. Meditation research may benefit from measuring dietary practices and/or modification which may impact (enhance, reduce) the treatment response of meditation practices.

### 4.5 Limitations

The present study has significant limitations, consistent with the early stage of this research. First, qualitative results are descriptive and cannot establish causal relationships. The hypotheses derived from the present study should be tested by future studies that quantitatively measure relationships between dietary changes and the incidence of and recovery from meditation-related challenges. Further research is needed to determine whether dietary changes indeed function as a risk factor, indicator of more severe symptomology, or remedy for meditation-related challenges. Second, the present findings include a wide range of meditation-related challenges, practices, and practice traditions, in addition to many different types of dietary changes. Future research may benefit from narrowing the scope of investigation as certain forms of dietary changes may impact certain forms of meditation-related challenges or certain practices/practice traditions. Studies with a narrower scope may also be able to identify more precise mechanisms. Third, the semi-structured interviews did not specifically query the role of diet in participants’ meditation-related challenges. As a result, participants may have left out important information regarding the role of dietary changes in their interviews. Finally, the present sample does not generalize to all meditation practitioners or all individuals who experience meditation-related challenges, as participants were Western Buddhists who reported meditation-related challenges and were primarily highly experienced, educated and White. Future studies should investigate the role of dietary changes in meditators with a different set of demographic characteristics to determine the level of generalizability of these findings.

## 5 Conclusion

In describing the various ways in which dietary changes play a role in the incidence, symptomology, and recovery from meditation-related challenges, the present study highlights the importance of considering diet-related factors when researching both risk factors and remedies for meditation-related challenges. Our findings suggest that a significant reduction in food intake, even if considered normative in one’s practice context, may contribute to the onset and trajectory of meditation-related challenges. Furthermore, diet-related remedies were described as an effective remedy or early intervention for meditation-related challenges. Finally, an overarching theme was the contrast between challenging or destabilizing “ungrounded” states resulting from intensive meditation and dietary restrictions and the remedial “grounding” effects of certain forms of dietary intake or the act of eating itself. Overall, this research highlights the need for increased attention to dietary practices as an important support for individuals and/organizations engaging in contemplative practices.

## Data Availability

The original contributions presented in the study are included in the article/supplementary material, further inquiries can be directed to the corresponding author.
